# Inside and outside the body space: a study on environmental knowledge, interoceptive and functional body representations in the prodromal stage of dementia

**DOI:** 10.1007/s10072-026-09294-9

**Published:** 2026-08-08

**Authors:** Fabiana Mogavero, Gaia Galluzzi, Erica Dolce, Daniela Malangone, Simona Raimo, Antonella Di Vita, Eleonora Galosi, Giuseppe Bruno, Micaela Sepe Monti, Giuseppina Talarico, Fabrizia D’Antonio, Liana Palermo

**Affiliations:** 1 Department of Human Neuroscience, Sapienza University of Rome, Rome, Italy; 2 Department of Medical and Surgical Sciences, Magna Graecia University of Catanzaro, Catanzaro, Italy; 3 Present Address: Department of Psychology, Sapienza University of Rome, Rome, Italy; 4 Present Address: Department of Clinical and Experimental Medicine, University of Messina, Messina, Italy

**Keywords:** Alzheimer’s disease, Mild cognitive impairment, Interoception, Spatial navigation, Body representation, Cognitive biomarkers

## Abstract

**Background:**

There is increasing interest in identifying sensitive cognitive markers for the early detection of Alzheimer’s disease (AD). Spatial navigation has emerged as a promising marker, as impairments in both egocentric and allocentric navigation have been reported in preclinical AD and mild cognitive impairment (MCI). However, findings regarding the relative involvement of these navigational representations remain inconsistent. Moreover, little is known about how MCI affects other forms of spatial representation beyond navigation.

**Objective:**

The present study aims to investigate navigational, inner, and outer bodily representations in individuals with MCI to provide a broader characterization of the cognitive changes associated with this condition across different bodily and navigational space representations.

**Methods:**

We conducted a cross-sectional comparative study involving 28 healthy controls (HC) and 25 participants diagnosed with MCI (PtwMCI). All participants completed tasks and questionnaires probing: (a) different interoceptive dimensions (interoceptive accuracy [IAcc], awareness [IAw], and sensibility [ISe]); (b) action-oriented (aBR) and nonaction-oriented (NaBR) body representations; (c) landmark, route, and survey knowledge of a virtual environment.

**Results:**

PtwMCI showed reduced IAcc and ISe and performed less accurately in landmark and route tasks compared to HC.

**Conclusions:**

Our findings indicate early impairments in interoceptive (inner body) and navigational processing, even in the initial stages of cognitive decline. These difficulties may thus represent an early marker of neurodegeneration. The observed co-occurrence of alterations across these domains highlights the need for future studies to clarify the relationship between body-related cognitive processes and broader cognitive functions in the Alzheimer’s disease continuum.

**Supplementary Information:**

The online version contains supplementary material available at https://doi.org/10.1007/s10072-026-09294-9.

## Introduction

Alzheimer’s Disease (AD) is the most common form of dementia worldwide, characterised by specific neuropathological changes and a gradual decline in cognitive, behavioural, and functional abilities [1].

Extensive research has focused on identifying specific biomarkers during the presymptomatic phase to enable early diagnosis of the disorder [2]. Several studies have also examined the neuropsychological impairment in the earliest stages of the disease. Patients with AD typically exhibit a distinctive pattern of cognitive decline predominantly involving episodic memory [3]. This pattern aligns with evidence indicating that the medial temporal lobe, particularly the perirhinal and hippocampal regions, is among the brain regions most susceptible to the initial neuropathological alterations associated with AD [4]. Although episodic memory impairment is widely recognised as one of the most robust and predictive cognitive markers of conversion to AD in its prodromal stages, its diagnostic specificity remains limited, as similar impairments are also observed in a range of other neurodegenerative disorders [5]. Consequently, there is growing interest in identifying additional cognitive markers as promising hallmarks for the early identification of individuals at risk of developing dementia. Spatial navigation abilities are gaining recognition as a cost-effective cognitive marker for identifying AD in its preclinical stages [6–7]. Indeed, navigational difficulties involving egocentric and allocentric representations of the environment have been described in preclinical AD and MCI [8–10]. Egocentric navigation relies on route-based representations anchored to the individual’s body, whereas allocentric navigation involves survey-based representations that encode spatial relationships between landmarks independently of the observer’s position [7]. Findings regarding the relative involvement of egocentric and allocentric strategies in preclinical and prodromal AD remain inconsistent. Allison and colleagues [8] reported selective impairments in allocentric wayfinding in individuals at preclinical stages of AD, whereas Weniger and colleagues [10] observed pronounced deficits in egocentric navigation and route-based strategies in patients with amnestic MCI.

Fewer studies have examined the effects of AD and MCI on other spatial representations. Following the conceptual distinction proposed by Tversky and colleagues [11], different types of space can be identified: the navigational space, the space around the body, and the body space—the latter being experienced both internally, through interoception, and externally, through exteroception.

Regarding the representation of the body from the inside, interoception, the sense of the physiological condition of the body [12], at the conscious level, can be operationalised along three main dimensions: (i) interoceptive accuracy (IAcc; the performance on objective tasks, such as heartbeat counting task); (ii) interoceptive sensibility (ISe; the self-perceived tendency to focus on interoceptive signals, measured using questionnaires); (iii) interoceptive awareness (IAw; the metacognitive awareness of IAcc, that is the correspondence between objective IAcc and subjective report) [13].

Few studies have examined interoceptive processing in AD [14, 15], reporting alterations in IAcc and IAw. Evidence in prodromal stages of AD remains even more limited; however, emerging neuroimaging findings point to alterations in interoceptive network connectivity associated with early cognitive decline [16]. Interestingly, some authors have associated interoceptive alterations in ageing with mitochondrial dysfunction, suggesting a possible link between impaired cellular energy metabolism and disrupted processing of internal bodily signals [17]. Given accumulating evidence linking mitochondrial dysfunction to AD pathogenesis [18], evaluating interoceptive processing in AD and its preclinical stages could be particularly promising.

Regarding the representation of the body from the outside, although different taxonomies have been proposed, a functional differentiation between action-oriented body representations (aBR), or body schema, a body representation that is tightly coupled with the space immediately surrounding the body (i.e., peripersonal space), and a nonaction-oriented body representation (NaBR), which includes a topological map of the body, is almost uncontroversial [19]. Studies across the lifespan suggest that both aBR and NaBR improve during childhood and decline in older age, following inverted U-shaped trajectories [20]. Studies on pathological ageing that have assessed aBR using mental rotation of body parts and motor imagery tasks indicate that MCI [21] and AD patients [22] show longer response times in hand mental rotation and body-centred motor imagery tasks, while accuracy remains largely preserved. Regarding the topological map of the body (NaBR), evidence is sparse. Still, a study of three patients with AD suggests that it is preserved at least when investigated using a task probing tactile distance discrimination [23].

Crucially, although interoception, aBR, and NaBR are closely related [20, 24], they capture different aspects of bodily processing and rely on partially distinct neural networks [12, 19, 24]. Assessing these bodily dimensions within a unified framework may help determine which aspects of body processing are selectively affected by prodromal neurodegeneration.

Importantly, the study of interoceptive and functional body representations is relevant not only to determine whether bodily space is selectively impaired, but also because, within the framework of embodied cognition, cognitive processes are thought to be grounded in bodily experience and representation [25]. Accordingly, growing evidence suggests that body representations play a pivotal role in cognitive abilities spanning from social cognition to memory and navigation [25–31]. Indeed, navigation entails acting with the body in the environment [30]. Consistent with this view, Tversky [31] proposed that the body represents the first space we come to know, even before birth, and serves as the primary medium through which we perceive and act upon other spaces. Within this perspective, functional body representations may support actions in space and the updating of one’s position and orientation during wayfinding.

Notably, current research is increasingly targeting interoceptive representations as particularly relevant to cognition. Specifically, a recent interpretative framework suggests that the processing of visceral inputs could provide a foundation for cognition by contributing to the construction of a first-person, body-centered perspective that supports subjective experience and cognitive functions [28], including navigation [26]. Furthermore, interoceptive bodily signals have been suggested to contribute to self-location [32], the experience of where one is in space, which is a prerequisite for navigation [33]. Consequently, alterations in interoceptive processing may contribute to spatial navigation deficits by disrupting information that supports the construction of a unified bodily reference frame and self-location within the environment. This possibility is particularly relevant in MCI, where spatial navigation is among the earliest cognitive functions to decline [6–7].

Although evidence from young adults supports the role of body representations in cognition, research in normal and pathological ageing remains limited [29]. In particular, a comprehensive investigation of bodily and extrabodily spatial representations in the same sample of individuals with AD in its preclinical stages remains lacking.

Building on evidence that different types of space can be distinguished and grounded in the embodied cognition framework, this study aims to extend current findings by comparing healthy older adults with those diagnosed with MCI in their ability to process navigational space -including landmark, route-based egocentric and survey-based allocentric spatial representations- and the body spaces in terms of action-oriented body representation, topological body map and inner (interoceptive) body representations.

## Methods

### Participants

Twenty-five participants with MCI (PtwMCI; 14 females, age: *M* = 70.50 yrs; *SD* = 4.78), diagnosed according to clinical criteria of the National Institute on Aging-Alzheimer’s Association (NIA-AA) [34–35], were recruited at the Cognitive Disorders and Dementia Center of “Policlinico Umberto I” Sapienza University Hospital of Rome (Italy). For all patients, clinical data, symptom onset, pharmacological therapy, neurological examination, and extensive neuropsychological assessment were collected. Patients with cognitive decline due to secondary causes (i.e., hydrocephalus, alcohol abuse, vitamin deficiency), a history of head trauma or psychiatric comorbidities, severe central nervous system infections, or a history of cerebrovascular diseases (i.e., cerebral haemorrhage, stroke, transient ischemic attacks) were excluded. Furthermore, because the experimental protocol required adequate comprehension of verbal instructions and questionnaires, patients with severe language impairment preventing task comprehension or completion of the self-report questionnaires were excluded a priori.

Based on the comprehensive clinical evaluation and the neuropsychological assessment battery administered for diagnostic purposes, all the PtwMCI group showed the expected pattern of reduced verbal episodic memory.

Beyond the expected impairment in verbal episodic memory, additional impairments in other cognitive domains were heterogeneous across participants, with no single domain showing a consistent pattern of impairment across the sample (see Supplementary Table S1 for representative neuropsychological findings for PtwMCI).

None of the PtwMCI showed evidence suggestive of lateralized attentional deficits based on their performance on the Visual Search Test and the Trail Making Test, although these measures provide only indirect evidence. Functional verbal comprehension, evaluated as part of the standard neuropsychological assessment, was preserved in all PtwMCI. Also, an item-level analysis of individual protocols showed that no patient failed the MMSE language comprehension items (see Supplementary Table S1).

Information on patients’ awareness of their cognitive deficits was retrospectively derived from the clinical judgment documented in the neuropsychological report. Based on this qualitative evaluation, awareness was considered preserved in 20 PtwMCI (80%), partially reduced in 3 PtwMCI (12%), and not specified in the reports for 2 PtwMCI (8%).

Participants were further classified as having amnestic single-domain (*n* = 7; 28%) or amnestic multiple-domain MCI (*n* = 18; 72%) based on the comprehensive clinical and neuropsychological evaluation, supported by neuroimaging findings as part of the diagnostic workup, according to the criteria proposed by the International Working Group on MCI [36].

Twenty-eight healthy controls (HC) (12 females, age: *M* = 68.90 yrs; *SD* = 4.55) were also recruited. None of them had a history of or current mental health disorders according to the Diagnostic and Statistical Manual of Mental Disorders, 5th Edition (DSM-5), or any neurological disease. All HC had normal scores at the MMSE (Italian cut-off > 23.8) [37].

All participants had normal or corrected-to-normal vision.

The sample size was based on previous studies on navigational memory in AD and its preclinical stages [10, 38]. A post hoc sensitivity power analysis (α = 0.05, 80% power) indicated that the present sample was sufficiently powered to detect medium-to-large effects (Cohen’s d ≥ 0.79).

The study was carried out in accordance with the Declaration of Helsinki, and Ethical approval was obtained from the local Ethics Committee. Informed written consent was obtained from all participants.

### Procedure

All participants completed tasks and questionnaires assessing the three different interoceptive dimensions (IAcc, IAw, and ISe), aBR, NaBR (i.e., the topological or visuo-spatial body map), and navigational abilities (landmark, route, and survey knowledge of a virtual environment).

For each of the aBR, NaBR, and navigational tasks, both accuracy and response time were recorded. Response times are reported in the supplementary material (Table S2).

### Evaluation of interoception

IAcc was evaluated using the Heartbeat Counting Task (HCT) [39]. It included six trials, each consisting of counting the number of heartbeats over three different temporal intervals (15, 20, and 18s), performed twice [26]. During the task, participants silently counted perceived heartbeats between start and stop signals, without taking their pulse, and were equipped with an optical heart rate sensor (Polar Verity Sense) placed on the arm to measure their actual heartbeats.

For each trial, an accuracy score was computed using the following formula: [1-(nbeatsreal-nbeatsreported)/((nbeatsreal+nbeatsreported/2)]; then, accuracy scores were averaged over six trials [13].

IAw was calculated by correlating IAcc with participants’ confidence in their ability to perceive heartbeats [13], rated at the end of each HCT trial on a 10-cm Visual Analogue Scale.

ISe was assessed using the Self-Awareness Questionnaire (SAQ) [40], which includes 35 items rated on a 5-point Likert scale, clustered into a visceral domain (ViD) and somatosensory domain (SoD). The total score ranges from 0 to 140, with higher scores indicating greater ISe.

Additionally, we employed theMultidimensional Assessment of Interoceptive Awareness (MAIA), a 32-item multidimensional instrument designed to measure awareness of body sensation, emotional response, attention regulation, and trust in bodily sensations [41]. MAIA uses a 5-point Likert scale, yielding a total score ranging from 0 to 140, with higher scores indicating greater ISe.

### Evaluation of aBR and NaBR

aBR was evaluated through the Hand Laterality Task (HLT) [42]. Participants were asked to decide on the laterality of a single hand drawing (48 stimuli: 24 left and 24 right), presented at varying degrees of angular rotation (0, 45, 90, 135, 180, 225, 270, and 315°) on the screen. The accuracy score corresponded to the sum of correct responses (range: 0–48), with higher scores indicating better performance.

NaBR was assessed using the computerised version of the Frontal Body Evocation task (FBE) [42]. Participants were shown an image of a body for 10 s, and then sequentially repositioned nine body parts on a touchscreen, placing each relative to the head, which served as the sole spatial reference. The computer recorded the coordinates of each placement before presenting the subsequent body part. Higher scores on the task reflect greater representational distortion and, therefore, a higher degree of error.

Response times are reported in the supplementary materials (Table S2).

### Evaluation of navigational abilities

Navigational abilities were assessed using a self-report questionnaire and objective behavioural computer-based navigation tasks.

Participants completed the “Santa Barbara Sense of Direction Scale” (SBSOD), which assesses the ability to orient oneself in the environment [43]. It includes 15 items to be rated on a 7-point Likert scale. The overall score is calculated as the mean of the 15 items. Scores range from 1 to 7, with higher scores indicating a better sense of direction.

Concerning the performance-based navigational tasks, we used three tasks designed to assess landmark, route, and survey knowledge (described in detail in reference [26]). First, participants carefully watched a video depicting a path through a virtual environment for approximately three minutes. The virtual environment was part of a navigational training program [44]. Then they performed the landmark, route, and survey tasks.

#### *Landmark task*

**Fig. 1:**
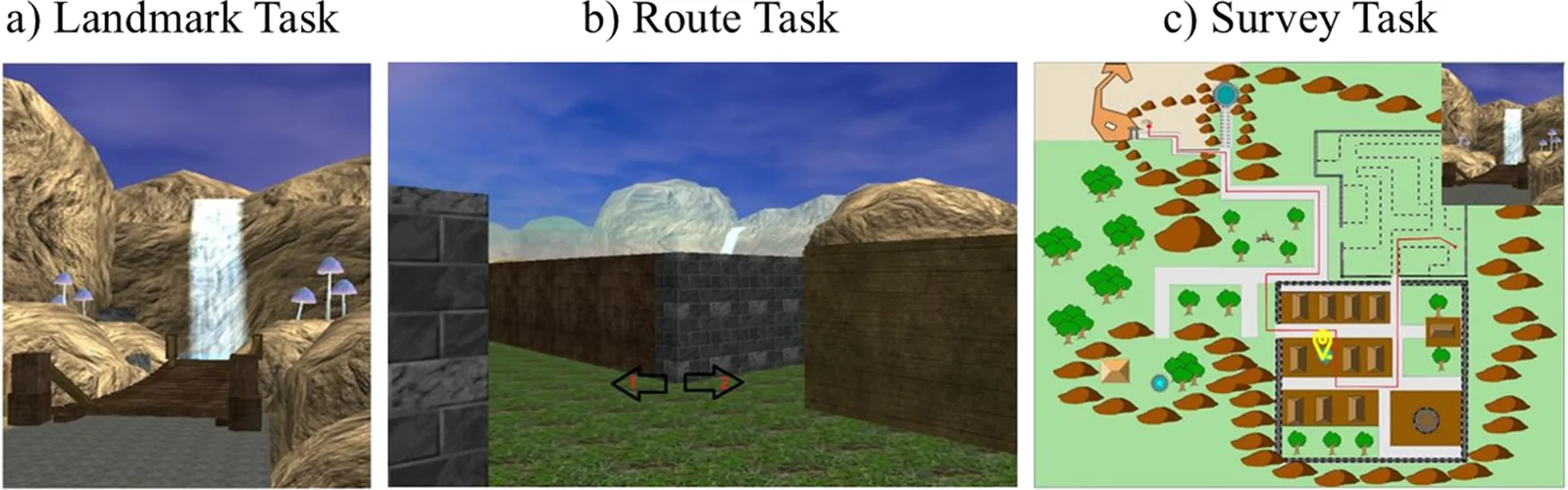
Stimuli from a computerised battery for evaluating navigational abilities. **a**) Landmark, **b**) route, and **c**) survey knowledge tasks

Participants were shown seven pictures of landmarks encountered along the path (e.g., a bridge) and seven pictures of distractors (e.g., a mountain hut). For each image, they were required to identify whether it represented a landmark seen during the navigation phase or a distractor [26] (maximum score: 14) (Fig. 1a).

#### *Route task*

Participants were presented with eight images taken from the navigated path, each of which displayed two possible continuation directions, marked by arrows labelled “1” and “2” (Fig. 1b). For every trial, participants were asked to select the correct direction (maximum score: 8) [26].

#### *Survey task*

Participants were shown an image of a landmark and a map of the virtual environment, in which a red line represented the path and a placeholder indicated the potential location of the landmark (Fig. 1c). Participants were asked to judge whether the landmark was correctly positioned on the map. The task comprised 14 trials (maximum score: 14) [26].

### Statistical analysis

Group differences on the main outcome measures were examined using independent-samples t-tests and chi-square (χ²) analyses. Only response accuracy was included in the statistical analyses; response times are reported in the Supplementary Material for completeness (see Table S2).

Exploratory Spearman’s rank correlation analyses were performed separately for the PtwMCI and HC groups to examine associations between body-related measures (IAcc, IAw, ISe, aBR, NaBR), navigational tasks (i.e., landmark, route, survey tasks) and cognitive status (MCI), in line with embodied cognition perspectives. Given the limited sample size, these analyses were considered exploratory, and full results are reported in the Supplementary Material (see Supplementary Tables S3 and S4).

No correction for multiple comparisons was applied to these exploratory analyses; therefore, the resulting associations should be interpreted with caution and regarded as preliminary findings.

## Results

### Between-group comparisons (HC vs. MCI)

Statistical analyses were conducted on 53 participants (28 HC and 25 MCI); descriptive statistics (means and standard deviations) and group differences are reported in Table 1.

**Table 1:** Descriptive statistics and between-group comparisons for demographic and cognitive measures (HC vs. PtwMCI)

	HC M (SD)	PtwMCI M (SD)	HC vs. PtwMCI		
			χ2/t	*p*	d
N	28	25	-	-	-
Sex (% female individuals)	22.60	26.40	0.91	0.339	-
Age (years)	68.90 (4.55)	70.50 (4.78)	-1.24	0.220	-
Education (years)	13.50 (4.62)	13.00 (4.28)	0.80	0.710	-
MMSE	28.50 (1.50)	26.50 (2.52)	3.14	.003**	-
IAcc	0.66 (0.29)	0.30 (0.49)	2.99	.005**	0.94
IAw	0.10 (0.52)	0.15 (0.42)	-0.32	0.754	− 0.09
MAIA	20.20 (6.08)	16.40 (4.09)	2.56	0.013*	0.71
SAQ	28.10 (14.60)	15.60 (13.80)	3.21	0.002**	0.88
aBR (CR)	18.60 (2.36)	17.60 (4.40)	1.13	0.262	0.31
NaBR (mm of dev.)	87.30 (50.70)	109 (47.10)	-1.63	0.110	− 0.44
Landmark (CR)	12.60 (1.34)	10.90 (2.31)	3.30	0.002**	0.90
Route (CR)	5.64 (1.34)	4.64 (1.47)	2.60	0.012*	0.71
Survey (CR)	9.36 (2.09)	8.24 (2.40)	1.81	0.076	0.49
SBSOD	4.61 (1.18)	3.70 (0.94)	3.12	0.003**	0.85

*HC* Healthy Controls, *PtwMCI *Participants with Mild Cognitive Impairment, *N* group size, *M* mean, *SD* standard deviation, *IAcc* Interoceptive Accuracy, *IAw* Interoceptive Awareness, *MAIA *Multidimensional Assessment of Interoceptive Awareness, *SAQ* Self-Awareness Questionnaire, *aBR* Action-oriented Body Representation, *NaBR* Nonaction-oriented Body Representation, *CR* correct responses, *mm of dev.* millimetres of deviation from corrected location (higher scores indicate worse performance), *SBSOD* Santa Barbara Sense of Direction Scale

Group differences were assessed using independent-samples t tests or chi-square (χ²) tests. Significant differences are in bold. *p < .05; **p <.01; d = Cohen’s d effect size

The comparison between the two groups revealed no differences in sex distribution (χ² = 0.913, *p* = .339), age (t = − 1.242, *p* = .22), or education (t = 0.375, *p* = .71). Significant differences emerged in MMSE performance (t = 3.141, *p* = .003), with the HC group outperforming the PtwMCI group.

Regarding interoceptive dimensions, significant group differences were observed in ISe (SAQ: t = 3.212, *p* = .002; MAIA: t = 2.563, *p* = .013) and IAcc (t = 2.993, *p* = .005), both of which were reduced in the PtwMCI group, whereas no statistically significant differences were found in IAw (t = − 0.315, *p* = .754).

Concerning the outer body representation related to action and surrounding body space (aBR) and the nonaction-oriented topological map of the body (NaBR), no significant differences were observed between the two groups (aBR: t = 1.134, *p* = .262; NaBR: t = − 1.627, *p* = .11).

Regarding the navigational space representations, the PtwMCI group performed worse than the HC group in the landmark (t = 3.296, *p* = .002) and route tasks (t = 2.6, *p* = .012), whereas no statistically significant difference emerged in the survey task (t = 1.809, *p* = .076). For the self-report measures, the PtwMCI group reported a poorer sense of direction compared to the HC group on the SBSOD (t = 3.119, *p* = .003).

### Spearman’s rank correlation

The exploratory analysis of associations between variables conducted on the PtwMCI group (see Table S3 in Supplementary Materials for full results) showed moderate positive correlations between route knowledge and aBR (rho = 0.537, *p* = .006), and between survey knowledge and SAQ (rho = 0.474, *p* = .017). No statistically significant associations were observed between bodily or navigational measures and MMSE scores.

The exploratory analysis conducted on the HC group revealed a negative association between route knowledge and NaBR (rho = -0.381, *p* = .046), that is, the better the performance in the route task, the fewer the mm of deviation from the correct location in the FBE task (see Table S4 in Supplementary Materials).

No other statistically significant correlations were observed among the variables in both groups.

## Discussion

Overall, within the framework of neuropsychological markers, this work highlights the importance of assessing interoceptive and navigational abilities for understanding early dysfunction in the prodromal stages of neurodegenerative disease. Consistent with previous literature (for an overview, see reference [7]), participants with MCI showed impaired landmark and route knowledge, suggesting deficits in creating an egocentric representation of the environment and sequential spatial processing. This finding reinforces the sensitivity of navigational measures in detecting subtle functional deficits even at mild levels of impairment.

Contrary to our hypothesis, participants with MCI did not exhibit significant impairment in survey knowledge, despite showing clear deficits in landmark- and route-based navigational tasks. The lack of significant group differences in survey knowledge is noteworthy and warrants careful interpretation. One possible explanation is that survey knowledge, which relies on the recognition of global spatial configurations, may place lower demands on episodic memory processes than landmark and route tasks, which require sequential recall and order memory, functions known to be particularly involved in hippocampal pathology during the early stages of MCI [7]. Moreover, the nature of the survey task may allow for greater reliance on compensatory strategies, such as encoding the relative locations of salient landmarks on a map, or may promote increased engagement of other brain regions, such as the parietal and frontal cortices. Such mechanisms could support preserved performance in the presence of hippocampal dysfunction. Alternatively, the absence of significant group differences may reflect the survey knowledge task’s intrinsic difficulty, thereby reducing its sensitivity to subtle group-level impairments. In addition, evidence from virtual environment studies indicates differential age-related trajectories in spatial navigation during physiological ageing, with egocentric strategies remaining relatively preserved and allocentric processing showing a progressive decline with ageing [7, 45]. Given that the MCI and control groups in the present study were closely matched for age, age-related reductions in allocentric spatial processing may have been present in both groups, potentially attenuating between-group differences in survey knowledge and limiting the ability of this measure to detect MCI-specific impairments. However, some methodological considerations are worth noting. Specifically, the absence of significant group differences may reflect the limited sensitivity of the task and/or the fact that the present study was powered to detect medium-to-large, but not smaller, effects.

Also, individuals with MCI reported greater navigational difficulties in self-report questionnaires. Notably, these subjective difficulties are broadly consistent with the difficulties reported in objective navigation tasks, suggesting convergence between self-report and behavioural measures.

Importantly, although the navigational tasks used in this study inevitably recruit other cognitive abilities, including episodic memory, attention, working memory, and executive functions, the relatively selective pattern of impairment observed across navigational, interoceptive, and functional body representation measures is unlikely to be explained solely by generalized cognitive decline. Indeed, despite the expected impairment in verbal episodic memory, the neuropsychological profile of the PtwMCI was not characterized by generalized impairment across cognitive domains (see Supplementary Table S1). Moreover, participants showed preserved performance on some body representation measures (see discussion below on aBR, NaBR, and IAw) and survey navigation.

Concerning the perception of one’s own bodily signals, our results suggest that MCI patients exhibited reduced interoceptive processing. Specifically, patients with MCI showed lower IAcc and ISe than healthy controls, while IAw appeared comparable across groups. These results extend previous findings on altered interoceptive processing in physiological and pathological ageing. A previous study [14] reported reduced IAcc in AD, and our findings indicate that similar alterations may already emerge during the prodromal stage. By contrast, IAw did not differ between groups; this dissociation aligns with the multidimensional model of interoception proposed by Garfinkel and colleagues [13], supporting the view that interoceptive dimensions rely on partially distinct neural mechanisms. The preservation of IAw in MCI may therefore reflect relatively intact cortical higher-order metacognitive processes, despite early degradation of bottom-up visceral signals, possibly related to insular or subcortical dysfunction [12].

Our results showed no significant differences between patients with MCI and HC in either aBR or NaBR accuracy, in line with previous literature [21, 23] and with evidence indicating that functional body representations may decline primarily in disorders with more pronounced sensorimotor involvement [19]. These findings suggest that higher-order functional body representations may remain relatively preserved in the prodromal stages of neurodegeneration, possibly because the frontoparietal neural networks supporting them [19] are only partially compromised in MCI.

Beyond group-level differences, exploratory correlational analyses provided preliminary insights into the associations among body-related representations, navigational processes, and cognitive status, in line with the current call for more research on embodied cognition in normal and pathological ageing [29]. The absence of associations with MMSE suggests that bodily and navigational measures are not simply related to global cognitive status. In contrast, the associations observed between functional body representations and route knowledge may point to a specific link between the body schema and egocentric navigation, which is consistent with embodied cognition perspectives. Importantly, given their exploratory nature and the absence of correction for multiple comparisons, these findings should be interpreted with caution and require replication in larger, adequately powered samples. Also, given the cross-sectional design of the present study, these data suggest co-occurring alterations across bodily and environmental spatial representations rather than a direct, directional, or causal relationship. Therefore, while the embodied cognition framework offers a valuable, integrative perspective for conceptualizing these findings, a more cautious interpretation is warranted.

Several limitations and opportunities for future research should be acknowledged. Regarding interoceptive assessment, while the ISe questionnaire assessed self-reported interoceptive experiences across multiple bodily domains, measures of IAcc and IAw were restricted to the cardiac domain. Also, although the HCT is one of the most widely used IAcc measures, its validity has been questioned, as performance on this task may partly rely on heart rate estimation [46]. Future studies should extend the assessment of interoception beyond the cardiac domain by considering multiple bodily systems (e.g., cardiac, gastric, and respiratory) and by employing additional measures of IAcc.

Another limitation is that spatial navigation was assessed using computer-based tasks, which, unlike real-world navigation, do not incorporate vestibular, proprioceptive, or motor information. Consequently, the extent to which the present findings generalise to navigation in real environments remains to be determined.

While our study focused on MCI as a prodromal stage of AD, future work should examine whether the deficits identified here hold in established AD and whether they are specific to the Alzheimer’s disease continuum or extend to other forms of dementia (e.g., Lewy body dementia or frontotemporal dementia). Also, longitudinal studies are needed to determine whether the observed deficits can predict progression from MCI to AD.

A dedicated, standardized measure of general awareness was not administered; information on patients’ self-awareness was retrospectively derived from clinical judgment documented in the neuropsychological report. Based on this classification, awareness appeared broadly preserved in the majority of PtwMCI, a pattern tentatively consistent with the preserved interoceptive awareness (IAw) observed at the group level; however, because this classification was based on clinical judgment rather than a validated measure, the potential influence of reduced awareness on self-reported measures cannot be ruled out. Future studies should employ validated, dedicated measures of general awareness/anosognosia.

Moreover, the present sample included only patients with amnestic MCI; future studies should extend these findings to non-amnestic MCI subtypes, in which the relationships among interoceptive, body-representation, and navigational impairments may differ.

In conclusion, despite some limitations, this study provides evidence of interoceptive alteration and navigation dysfunction in prodromal dementia. However, further research is needed to clarify the relationship between these domains.

## Supplementary Information

Below is the link to the electronic supplementary material.

Supplementary Material 1 (DOCX 482 KB)

## Data Availability

All data supporting this research are publicly available. Link: https://osf.io/57x8w.
