# Supplementary material for: Inside and outside the body space: a study on environmental knowledge, interoceptive and functional body representations in the prodromal stage of dementia
Source: Neurol Sci. 2026 Aug 8;47(9):692. doi: 10.1007/s10072-026-09294-9 (PMC13451470; doi:10.1007/s10072-026-09294-9)
Supplement: Supplementary file 1 [file 10072_2026_9294_MOESM1_ESM.docx]

**Supplemental Material**

**Supplementary Table S1. |** Summary of representative neuropsychological test results for participants with Mild Cognitive Impairment.

| ID | Awareness | Verbal Comprehension (functional) | Verbal Comprehension (MMSE) | Episodic Memory-verbal (RAVLT) | Episodic Memory-verbal (Prose Memory Test) | Episodic Memory-Visuospatial (ROCFr) | Selective Attention (Visual search) | Speed (TMT-A) | FAB | Visuospatial Reasoning (Raven’s PM) | Verbal WM (Digit Span BW) | Visuospatial WM (Corsi Span BW) | Visuoconstructive abilities  (ROCFc) |
| --- | --- | --- | --- | --- | --- | --- | --- | --- | --- | --- | --- | --- | --- |
| 1 | nci | nci | + | - | - | - | + | + | + | + | + | + | + |
| 2 | nci | nci | + | - | + | + | + | + | + | + | + | + | + |
| 3 | nci | nci | + | - | + | + | + | + | + | + | + | + | + |
| 4 | nci | nci | + | - | + | + | + | + | - | + | + | + | + |
| 5 | pci | nci | + | + | - | - | + | - | + | + | + | + | + |
| 6 | nci | nci | + | - | - | + | + | + | + | + | + | + | + |
| 7 | ns | nci | + | - | + | - | + | + | + | + | + | + | + |
| 8 | nci | nci | + | - | + | + | + | + | + | + | + | + | + |
| 9 | nci | nci | + | - | + | + | + | + | + | + | + | + | + |
| 10 | nci | nci | + | - | + | + | + | + | + | + | + | + | + |
| 11 | nci | nci | + | - | + | - | + | + | + | + | + | + | - |
| 12 | nci | nci | + | - | + | + | + | + | + | + | + | + | + |
| 13 | nci | nci | + | - | + | - | + | + | + | + | + | + | + |
| 14 | nci | nci | + | + | - | + | + | + | - | + | + | - | + |
| 15 | nci | nci | + | - | + | + | + | + | + | + | - | + | + |
| 16 | nci | nci | + | - | + | + | + | + | - | + | + | + | + |
| 17 | ns | nci | + | - | - | + | + | + | + | + | + | + | + |
| 18# | nci | nci | + | + | + | + | + | + | + | + | + | + | + |
| 19## | nci | nci | + | + | + | + | + | + | + | + | + | + | + |
| 20 | nci | nci | + | - | + | - | + | + | - | + | na | + | + |
| 21 | nci | nci | + | - | + | + | + | + | + | + | + | + | + |
| 22 | nci | nci | + | - | - | - | - | - | - | - | + | + | - |
| 23 | nci | nci | + | - | + | + | + | + | - | + | + | + | + |
| 24 | pci | nci | + | - | - | - | + | + | - | + | + | + | + |
| 25 | pci | nci | + | - | - | + | + | + | + | + | + | + | + |

Table S1 summarizes selected results from the clinical and neuropsychological assessment performed for diagnostic purposes.

The reported tests are representative of the main cognitive domains and include: language comprehension (MMSE comprehension subtests: Folstein, 1975; Magni et al., 1996), verbal episodic memory (Prose Memory Test: Spinnler & Tognoni, 1987; and RAVLT-Rey Auditory Verbal Learning Test: Carlesimo et al., 1996; Rey, 1958), visuo-spatial episodic memory (ROCFr-Rey–Osterrieth Complex Figure recall: Spinnler & Tognoni, 1987), verbal working memory (Digit Span backward (BW): Orsini et al., 1987), visuospatial working memory (Corsi Block-Tapping Test backward (BW): Corsi, 1972; Spinnler & Tognoni, 1987; Monaco et al., 2013), visuoconstructive abilities (ROCFc-Rey–Osterrieth Complex Figure copy: Spinnler & Tognoni, 1987), selective attention (Visual Search Test: Spinnler & Tognoni 1987), speed of processing (TMT-A, Trail Making Test Part A: Reitan, 1958; Giovagnoli et al., 1996), global executive functioning (FAB-Frontal Assessment Battery: Dubois et al., 2000; Apollonio et al., 2005), and visuospatial reasoning (Raven's Progressive Matrices (PM): Basso, Capitani, & Laiacona, 1987; Raven, 1938).

For each participant, performance is classified as within the normal range (+) or impaired (−) according to the normative values of the selected neuropsychological tests (see the supplementary references for details).

The clinical evaluation of verbal comprehension is classified as no clinical impairment (nci), partial clinical impairment (pci), or clinical impairment (ci).

Patients' awareness of their cognitive deficits was retrospectively derived from the clinical judgment documented in the neuropsychological report and classified as no clinical impairment (nci), partial clinical impairment (pci), or clinical impairment (ci).

**Note:** + Performance within the normal range; - Impaired performance; nci: no clinical impairment; pci: partial clinical impairment; ns: Not specified in the clinical report; na: Data not available.

#For Patient 18, performance on the episodic memory tests was at the lower limit of the normal range. The overall clinical evaluation, supported by the neuropsychological assessment and neuroimaging findings obtained during the diagnostic workup, was consistent with a memory impairment.

##For Patient 19, the memory deficit was identified during a more comprehensive clinical assessment of memory function using the Rivermead Behavioural Memory Test–3 (RBMT-3; Wilson et al, 2013).

**Supplementary Table S2**. Descriptive statistics for response times on the navigation (Landmark, Route, Survey), action-oriented and nonaction-oriented body representation tasks in healthy controls (HC) and participants with Mild Cognitive Impairment (PtwMCI).

|  | Group | Landmark | Route | Survey | NaBR (FBE) | aBR (HLT) |
| --- | --- | --- | --- | --- | --- | --- |
| N | HC | 28 | 28 | 28 | 28 | 28 |
|  | PtwMCI | 25 | 25 | 25 | 24 | 25 |
| M | HC | 37.4 | 41.9 | 123 | 54.4 | 71.8 |
|  | MCI | 61.9 | 67.2 | 208 | 75.6 | 110 |
| Median | HC | 34.7 | 40.6 | 121 | 46.3 | 47.2 |
|  | PtwMCI | 55.8 | 61.5 | 195 | 61.4 | 77.3 |
| SD | HC | 16.6 | 16 | 40.7 | 21.4 | 58.3 |
|  | PtwMCI | 27.9 | 32.4 | 89.9 | 35.3 | 73.8 |
| Min | HC | 16.6 | 5.93 | 15.9 | 16.3 | 26.9 |
|  | PtwMCI | 29.8 | 23.5 | 82.4 | 29.1 | 32.4 |
| Max | HC | 96.7 | 77.1 | 185 | 107 | 246 |
|  | PtwMCI | 137 | 158 | 461 | 152 | 312 |

**Note**: For each measure, the table reports sample size (N), mean (M), median, standard deviation (SD), and minimum and maximum total response times in seconds (s) for both groups. NaBR = Nonaction-oriented Body Representation; FBE: Frontal Body Evocation; aBR = Action-oriented Body Representation; HLT: Hand Laterality Task*.*

**Supplementary Table S3. |** Spearman's rank correlations between interoceptive measures, aBR, NABR, and navigational tasks in the PtwMCI group.

| PtwMCI group Spearman’s rank correlation | | | | | | | | |
| --- | --- | --- | --- | --- | --- | --- | --- | --- |
|  |  | IAcc | IAw | ISe (MAIA) | ISe (SAQ) | NaBR | aBR | MMSE |
| Landmark | ρ | 0.093 | 0.429 | -0.211 | 0.023 | 0.128 | 0.222 | 0.361 |
|  | (*p*) | 0.722 | 0.086 | 0.322 | 0.914 | 0.543 | 0.287 | 0.076 |
| Route | ρ | -0.369 | 0.128 | -0.192 | 0.229 | -0.327 | 0.537** | -0.132 |
|  | (*p*) | 0.145 | 0.624 | 0.369 | 0.271 | 0.111 | 0.006 | 0.529 |
| Survey | ρ | -0.185 | 0.361 | -0.183 | 0.474* | -0.268 | 0.246 | -0.000 |
|  | (*p*) | 0.476 | 0.155 | 0.393 | 0.017 | 0.195 | 0.236 | 0.999 |
| SBSOD | ρ | 0.185 | 0.225 | 0.355 | -0.008 | -0.056 | 0.024 | 0.307 |
|  | (*p*) | 0.476 | 0.386 | 0.089 | 0.969 | 0.791 | 0.91 | 0.136 |
| MMSE | ρ | -0.017 | 0.210 | 0.118 | 0.115 | -0.120 | 0.077 | - |
|  | (*p*) | 0.947 | 0.420 | 0.584 | 0.569 | 0.569 | 0.715 | - |

**Note:** IAcc = Interoceptive Accuracy; IAw = Interoceptive Awareness; ISe =Interoceptive sensibility; MAIA = Multidimensional Assessment of Interoceptive Awareness; SAQ = Self-Awareness Questionnaire; aBR = Action-oriented Body Representation; NaBR = Nonaction-oriented Body Representation; MMSE: Mini-Mental State Examination; SBSOD = Santa Barbara Sense of Direction Scale; ρ = Spearman’s rank correlation coefficient; *p < .05; **p < .01.

**Supplementary Table S4. |** Spearman's rank correlations between interoceptive measures, aBR, NABR, and navigational tasks in the HC group.

| HC group Spearman’s rank correlation | | | | | | | | |
| --- | --- | --- | --- | --- | --- | --- | --- | --- |
|  |  | IAcc | IAw | ISe (MAIA) | ISe (SAQ) | NaBR | aBR | MMSE |
| Landmark | ρ | -0.092 | 0.376 | 0.118 | -0.294 | -0.335 | 0.003 | 0.299 |
|  | (*p*) | 0.661 | 0.064 | 0.551 | 0.128 | 0.081 | 0.986 | 0.176 |
| Route | ρ | 0.155 | -0.124 | -0.048 | -0.024 | -0.381* | 0.103 | 0.279 |
|  | (*p*) | 0.459 | 0.554 | 0.809 | 0.902 | 0.046 | 0.602 | 0.209 |
| Survey | ρ | 0.077 | 0.391 | 0.072 | -0.224 | -0.079 | 0.295 | -0.145 |
|  | (*p*) | 0.716 | 0.053 | 0.715 | 0.251 | 0.691 | 0.127 | 0.519 |
| SBSOD | ρ | 0.244 | 0.392 | 0.344 | -0.19 | 0.22 | 0.157 | 0.322 |
|  | (*p*) | 0.24 | 0.053 | 0.073 | 0.333 | 0.261 | 0.424 | 0.144 |
| MMSE | ρ | -0.034 | 0.198 | 0.011 | -0.385 | -0.067 | 0.05 | - |
|  | (*p*) | 0.881 | 0.418 | 0.965 | 0.077 | 0.768 | 0.824 | - |

**Note:** IAcc = Interoceptive Accuracy; IAw = Interoceptive Awareness; ISe =Interoceptive sensibility; MAIA = Multidimensional Assessment of Interoceptive Awareness; SAQ = Self-Awareness Questionnaire; aBR = Action-oriented Body Representation; NaBR = Nonaction-oriented Body Representation; MMSE: Mini-Mental State Examination; SBSOD = Santa Barbara Sense of Direction Scale; ρ = Spearman’s rank correlation coefficient. *p < .05; **p < .01.

**Supplementary References**

**Basso A, Capitani E, Laiacona M (1987) Raven's Coloured Progressive Matrices: normative values on 305 adult normal controls. Funct Neurol 2:189–194.**

Carlesimo GA, Caltagirone C, Gainotti G and the MDB Group (1996) The Mental Deterioration Battery: normative data, diagnostic reliability and qualitative analyses of cognitive impairment. Eur Neurol 36:378–384.

Dubois B, Slachevsky A, Litvan I, Pillon B (2000) The FAB: a Frontal Assessment Battery at bedside. Neurology 55:1621–1626.

Folstein MF, Folstein SE, McHugh PR (1975) Mini-mental state. A practical method for grading the cognitive state of patients for the clinician. J Psychiatr Res 12:189–198.

Giovagnoli AR, Del Pesce M, Mascheroni S, Simoncelli M, Laiacona M, Capitani E. (1996) Trail making test: normative values from 287 normal adult controls. Ital J Neurol Sci. Aug;17(4):305-9.

Magni E, Binetti G, Bianchetti A, Rozzini R, Trabucchi M (1996) Mini-Mental State Examination: a normative study in Italian elderly population. Eur J Neurol 3:198–202.

Monaco M, Costa A, Caltagirone C, Carlesimo GA. Forward and backward span for verbal and visuo-spatial data: standardization and normative data from an Italian adult population. Neurol Sci. 2013 May;34(5):749-54. doi: 10.1007/s10072-012-1130-x. Epub 2012 Jun 12. Erratum in: Neurol Sci. 2015 Feb;36(2):345-7. doi: 10.1007/s10072-014-2019-7. PMID: 22689311.

Orsini A, Grossi D, Capitani E, Laiacona M, Papagno C, Vallar G (1987) Verbal and spatial immediate memory span: normative data from 1355 adults and 1112 children. Ital J Neurol Sci 8:539–548.

Raven JC (1938) Progressive Matrices. London: H.K. Lewis.

Reitan RM (1958) Validity of the Trail Making Test as an indicator of organic brain damage. Percept Mot Skills 8:271–276.

Spinnler H, Tognoni G (1987) Standardizzazione e taratura italiana di test neuropsicologici. Ital J Neurol Sci Suppl 6(Suppl. 8), 1–20.

Wilson BA, Greenfield E, Clare L, Baddeley A, Cockburn J, Watson P, Tate R, Sopena S, Nannery R, Crawford J; adattamento italiano [Italian adaptation]: Beschin N, Urbano T, Treccani B (2013) Rivermead Behavioural Memory Test–Third Edition (RBMT-3). Firenze: Giunti O.S.
